# Supplementary material for: Development of a dose-response model for porcine cysticercosis
Source: PLoS One. 2022 Mar 14;17(3):e0264898. doi: 10.1371/journal.pone.0264898 (PMC8920259; doi:10.1371/journal.pone.0264898)
Supplement: S1 Table — (DOCX) [file pone.0264898.s001.docx]

**S1 Table. Standardized dose units for each exposure pathway.**

***Supplement to* “Development of a dose-response model for porcine cysticercosis”**

| **Exposure pathway** | **Original inoculum dose** | **Standardized inoculum dose** |
| --- | --- | --- |
| Proglottids | 0 proglottids | 0 eggs |
|  | ¼ proglottid | 10168 ± 1454.1 eggs |
|  | ½ proglottid | 18189 ± 3123.3 eggs |
|  | 1 proglottid | 39800 ± 5607.3 eggs |
| Beetles | 1 beetle | 28 ± 24.9 eggs |
|  | 3 beetles | 103 ± 43.5 eggs |
|  | 4 beetles | 113 ± 38.5 eggs |
|  | 6 beetles | 244 ± 96.8 eggs |
| Carotid | 2500 AO | 2500 eggs |
|  | 5000 AO | 5000 eggs |
|  | 10000 AO | 10000 eggs |
|  | 45000 AO | 45000 eggs |
|  | 50000 AO | 50000 eggs |

Legend: Proglottids: direct ingestion of gravid proglottids; Beetles: direct ingestion of beetles previously fed with eggs; Carotid: inoculation of activated oncospheres via catheterization of the common carotid artery; AO: activated oncospheres.
