# Supplementary material for: Development of a dose-response model for porcine cysticercosis
Source: PLoS One. 2022 Mar 14;17(3):e0264898. doi: 10.1371/journal.pone.0264898 (PMC8920259; doi:10.1371/journal.pone.0264898)
Supplement: S2 Table — (DOCX) [file pone.0264898.s002.docx]

**S2 Table. Cysticercosis challenge studies with references.**

***Supplement to* “Development of a dose-response model for porcine cysticercosis”**

| **Ref.** | **Exposure pathway** | **Dose** | **Exp** | **Inf_ac_** | **Inf_vc_** | **Inf_bc_** |
| --- | --- | --- | --- | --- | --- | --- |
| [1] | Controls | 0 proglottids/eggs | 3 | 0 | 0 | 0 |
|  | Proglottids | ¼ proglottids | 8 | 8 | 7 | 1 |
|  |  | ½ proglottids | 8 | 7 | 5 | 3 |
|  |  | 1 proglottids | 24 (14)^a^ | 24 | 23 | 7 |
|  | Eggs | 100 eggs | 4 | 3 | 1 | 0 |
|  |  | 1000 eggs | 4 | 4 | 2 | 0 |
|  |  | 10000 eggs | 4 | 4 | 4 | 0 |
| [2] | Proglottids | 1 proglottids | 6 | 4 | 4 | NA |
| [3] | Controls | 0 eggs | 4 | 0 | 0 | 0 |
|  | Eggs | 10 eggs | 4 | 3 | 1 | 0 |
|  |  | 100 eggs | 5 | 3 | 1 | 0 |
|  |  | 1000 eggs | 4 | 3 | 1 | 0 |
|  |  | 10000 eggs | 5 | 5 | 5 | 2 |
|  |  | 100000 eggs | 5 | 5 | 5 | 3 |
| [4] | Eggs | 20000 eggs | 5 | 5 | 5 | 4 |
| [5] | Beetles | 1 beetle | 6 | 5 | 4 | 1 |
|  |  | 3 beetles | 6 | 6 | 6 | 1 |
|  |  | 6 beetles | 6 | 6 | 6 | 2 |
| [6] | Beetles | 6 beetles | 16 | 14 | 13 | 0 |
| [7] | Carotid | 10000 AO | 6 | 4 | 4 | 3 |
|  |  | 45000 AO | 1 | 1 | 1 | 1 |
|  |  | 50000 AO | 5 | 5 | 5 | 4 |
| UD | Eggs | 1000 eggs | 3 | 3 | 0 | 0 |
|  | Beetles | 4 beetles | 8 | 6 | 2 | 0 |
|  |  | 6 beetles | 8 | 4 | 4 | 0 |
|  | Carotid | 2500 AO | 5 | 5 | 5 | 3 |
|  |  | 5000 AO | 6 | 6 | 6 | 6 |
|  |  | 10000 AO | 5 | 5 | 5 | 4 |

Legend: Exp: exposed pigs; Inf_ac_: infected pigs with any type of cyst; Inf_vc_: infected pigs with viable cysts; Inf_bc_: infected pigs with brain cysts; UD: unpublished data; Controls: negative controls; Proglottids: direct ingestion of gravid proglottids; Eggs: inoculation via an endoesophageal tube of eggs placed in a gelatin capsule; Beetles: direct ingestion of beetles previously fed with eggs; Carotid: inoculation of activated oncospheres via catheterization of the common carotid artery; AO: activated oncospheres; NA: not available.

^a^ Brain cyst counts were only available for 14 pigs.

**References**

1. Silva ME. Modelo de infección experimental oral para cisticercosis porcina por Taenia solium. National University of San Marcos. 2004.

2. Deckers N, Kanobana K, Silva M, Gonzalez AE, Garcia HH, Gilman RH, et al. Serological responses in porcine cysticercosis: A link with the parasitological outcome of infection. Int J Parasitol. 2008;38: 1191–1198. doi:10.1016/j.ijpara.2008.01.005

3. Santamaría E, Plancarte A, De Aluja AS. The experimental infection of pigs with different numbers of Taenia solium eggs: Immune response and efficiency of establishment. J Parasitol. 2002;88: 69–73. doi:10.1645/0022-3395(2002)088[0069:TEIOPW]2.0.CO;2

4. De Aluja AS, Villalobos NM, Nava G, Toledo A, Martínez JJ, Plancarte A, et al. Therapeutic capacity of the synthetic peptide-based vaccine against Taenia solium cysticercosis in pigs. Vaccine. 2005;23: 4062–4069. doi:10.1016/j.vaccine.2004.11.076

5. Gomez-Puerta LA, Garcia HH, Gonzalez AE. Experimental porcine cysticercosis using infected beetles with Taenia solium eggs. Acta Trop. 2018;183: 92–94. doi:10.1016/j.actatropica.2018.04.003.

6. Gonzales-Gustavson E. Detección de huevos de Taenia solium en el intestino de escarabajos coprófagos mediante PCR. National University of San Marcos. 2011. Available: http://cybertesis.unmsm.edu.pe/bitstream/handle/cybertesis/4147/Diaz_rc.pdf;jsessionid=CD5A7FF3022F1A5526948369A600356D?sequence=1

7. Alroy KA, Arroyo G, Gilman RH, Gonzales-Gustavson E, Gallegos L, Gavidia CM, et al. Carotid taenia solium oncosphere infection: A novel porcine neurocysticercosis model. Am J Trop Med Hyg. 2018;99: 380–387. doi:10.4269/ajtmh.17-0912
