# Supplementary material for: Development of a dose-response model for porcine cysticercosis
Source: PLoS One. 2022 Mar 14;17(3):e0264898. doi: 10.1371/journal.pone.0264898 (PMC8920259; doi:10.1371/journal.pone.0264898)
Supplement: S1 Appendix — (DOCX) [file pone.0264898.s004.docx]

**S1 Appendix. Assessment of the fit of the dose-response models.**

***Supplement to* “Development of a dose-response model for porcine cysticercosis”**

**Table A. Sum of Squared Errors of Prediction (SEE) for each dose-response model.**

| **Exposure pathway** | **Two-parameters log-logistic** | **Logistic regression** | **Exponential regression** | **Approximate beta-Poisson** | **Exact beta-Poisson** |
| --- | --- | --- | --- | --- | --- |
| **Development of any (viable or degenerated) cyst** | | | | | |
| Oral | NA | NA | NA | NA | 0.096 |
| Proglottids | NSP | 0.410 | NSP | 1.020 | 0.008 |
| Eggs | NA | 0.314 | 0.393 | 0.062 | 0.072 |
| Beetles | NA | 0.789 | NSP | 0.752 | 0.041 |
| Carotid | NA | 0.839 | NSP | 1.033 | 0.027 |
| **Development of viable cysts** | | | | | |
| Oral | NA | NA | NA | NA | 0.808 |
| Proglottids | NSP | 0.273 | 0.108 | 0.050 | 0.051 |
| Eggs | NSP | 0.037 | 0.097 | 0.164 | 0.164 |
| Beetles | NSP | NSP | 0.566 | 0.349 | 0.299 |
| Carotid | NA | 0.839 | NSP | 1.033 | 0.027 |
| **Development of brain cysts** | | | | | |
| Oral | NA | NA | NA | NA | 0.307 |
| Proglottids | NSP | 0.031 | 0.009 | 0.032 | 0.033 |
| Eggs | NSP | 0.456 | 0.219 | 0.199 | 0.199 |
| Beetles | NSP | NSP | NSP | 0.621 | 0.026 |
| Carotid | NSP | NSP | 0.186 | 0.292 | 0.134 |

Legend: Proglottids: direct ingestion of gravid proglottids; Eggs: inoculation via an endoesophageal tube of eggs placed in a gelatin capsule; Beetles: direct ingestion of beetles previously fed with eggs; Carotid: inoculation of activated oncospheres via catheterization of the common carotid artery; NSP: non-significant parameters; NA: non-developed model.

**Table B. Coefficient of Determination (R^2^) for each dose-response model.**

| **Exposure pathway** | **Two-parameters log-logistic** | **Logistic regression** | **Exponential regression** | **Approximate beta-Poisson** | **Exact beta-Poisson** |
| --- | --- | --- | --- | --- | --- |
| **Development of any (viable or degenerated) cyst** | | | | | |
| Oral | NA | NA | NA | NA | 0.893 |
| Proglottids | NSP | 0.191 | NSP | 0.258 | 0.988 |
| Eggs | NA | 0.625 | 0.752 | 0.928 | 0.918 |
| Beetles | NA | 0.152 | NSP | 0.335 | 0.929 |
| Carotid | NA | 0.110 | NSP | 0.184 | 0.965 |
| **Development of viable cysts** | | | | | |
| Oral | NA | NA | NA | NA | 0.527 |
| Proglottids | NSP | 0.333 | 0.840 | 0.913 | 0.911 |
| Eggs | NSP | 0.970 | 0.939 | 0.886 | 0.886 |
| Beetles | NSP | NSP | 0.468 | 0.563 | 0.557 |
| Carotid | NA | 0.110 | NSP | 0.184 | 0.965 |
| **Development of brain cysts** | | | | | |
| Oral | NA | NA | NA | NA | 0.612 |
| Proglottids | NSP | 0.808 | 0.939 | 0.761 | 0.752 |
| Eggs | NSP | 0.423 | 0.801 | 0.592 | 0.592 |
| Beetles | NSP | NSP | NSP | 0.502 | 0.210 |
| Carotid | NSP | NSP | 0.808 | 0.555 | 0.788 |

Legend: Proglottids: direct ingestion of gravid proglottids; Eggs: inoculation via an endoesophageal tube of eggs placed in a gelatin capsule; Beetles: direct ingestion of beetles previously fed with eggs; Carotid: inoculation of activated oncospheres via catheterization of the common carotid artery; NSP: non-significant parameters; NA: non-developed model.
