# Supplementary material for: Development of a dose-response model for porcine cysticercosis
Source: PLoS One. 2022 Mar 14;17(3):e0264898. doi: 10.1371/journal.pone.0264898 (PMC8920259; doi:10.1371/journal.pone.0264898)
Supplement: S2 Appendix — (DOCX) [file pone.0264898.s005.docx]

**S2 Appendix. Two-parameter log-logistic regression model.**

***Supplement to* “Development of a dose-response model for porcine cysticercosis”**

$$P_{inf}\left( D \right)=\frac{1}{1+exp\{\beta_{slope}[{log}_{e} \left( D \right)-{log}_{e}(\beta_{ID50})]\}}$$

**Table A. Parameter estimates for two-parameter log-logistic regression model.**

| **Exposure pathway** | **β_slope_** | **β_ID50_** |
| --- | --- | --- |
| **Development of any (viable or degenerated) cyst** | | |
| Proglottids | 0.72 (-0.37 – 0.38) | 9.27 x 10^5^ (-1.66 x 10^8^ – 1.68 x 10^8^) |
| Eggs | NA | NA |
| Beetles | NA | NA |
| Carotid | NA | NA |
| **Development of viable cysts** | | |
| Proglottids | -0.21 (-1.02 – 0.60) | 28.11 (-583.67 – 639.89) |
| Eggs | -1.86 (-7.38 – 3.67) | 1643.57 (-580.87 – 3868.01) |
| Beetles | 0.01 (-1.08 – 1.27) | 1.58 x 10^-5^ (-1.33 x 10^7^ – 1.36 x 10^7^) |
| Carotid | NA | NA |
| **Development of brain cysts** | | |
| Proglottids | -1.25 (-2.69 – 0.13) | 3.47 x 10^4^* (7990.99 – 6.14 x 10^4^) |
| Eggs | -0.77 (-1.84 – 0.31) | 1.96 X 10^4^ (-1.17 x 10^4^ – 5.09 x 10^4^) |
| Beetles | 0.56 (-1.66 – 2.77) | 1.57 (-21.02 – 24.16) |
| Carotid | -0.43 (-2.27 – 1.42) | 351.39 (-4248.89 – 4951.66) |

Legend: β_slope_, slope parameter; β_ID50_, median infective dose parameter; Proglottids, direct ingestion of gravid proglottids; Eggs, inoculation via an endoesophageal tube of eggs placed in a gelatin capsule; Beetles, direct ingestion of beetles previously fed with eggs; Carotid, inoculation of activated oncospheres via catheterization of the common carotid artery; NA, not available.

* p-value > 0.05


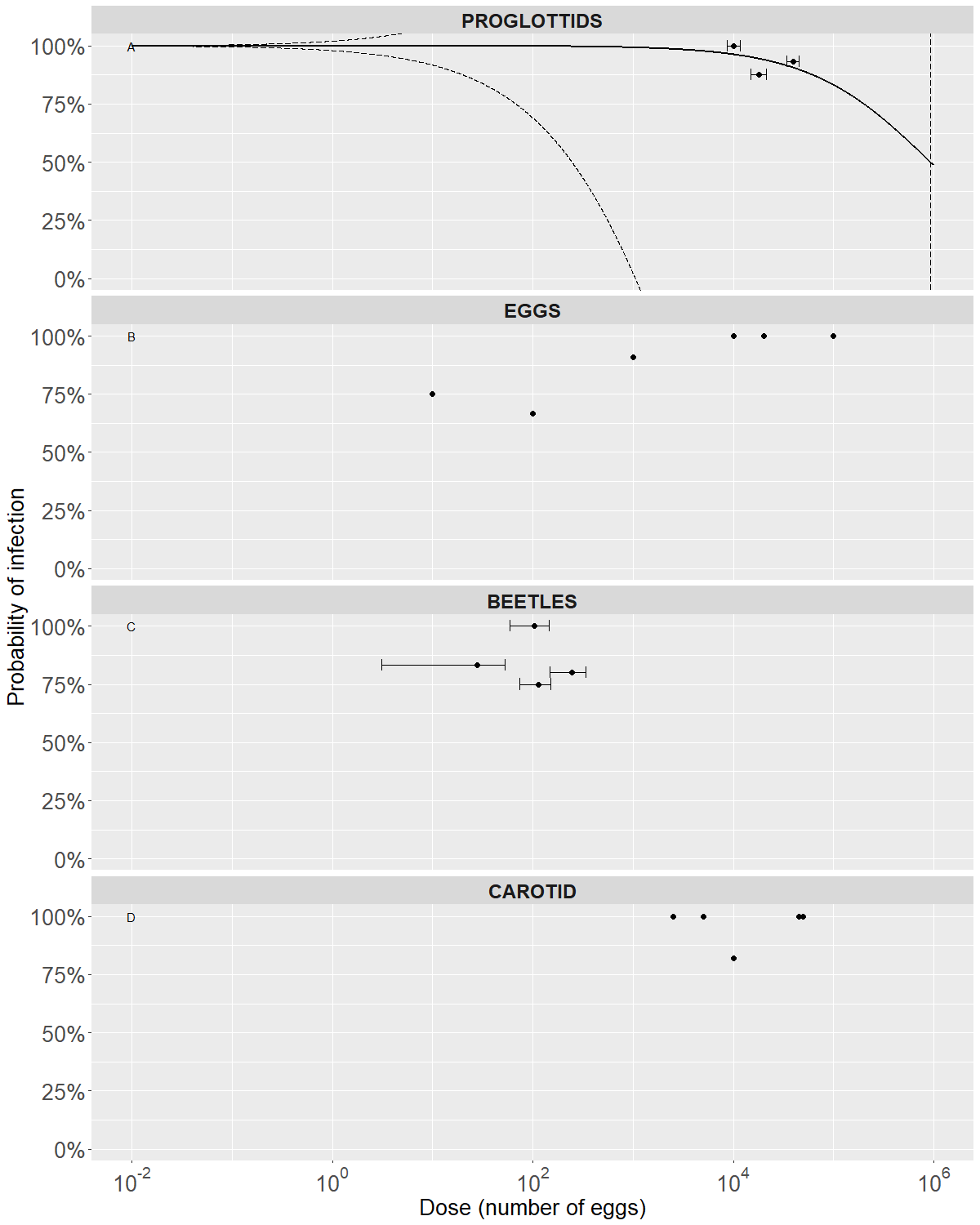


**Fig A. Two-parameter log-logistic regression dose-response relationship for the development of any cyst by exposure pathway.** Each graph shows the median (solid black curve) and 95% range (dashed black curves) of the probability of infection as a function of dose, median ID50 infectious dose (dashed black vertical line), and the available data point with its standard deviation (“Proglottids” and “Beetles” pathways only). (A) Direct ingestion of gravid proglottids. (B) Inoculation via an endoesophageal tube of eggs placed in a gelatin capsule. (C) Direct ingestion of beetles previously fed with eggs. (D) Inoculation of activated oncospheres via catheterization of the common carotid artery.


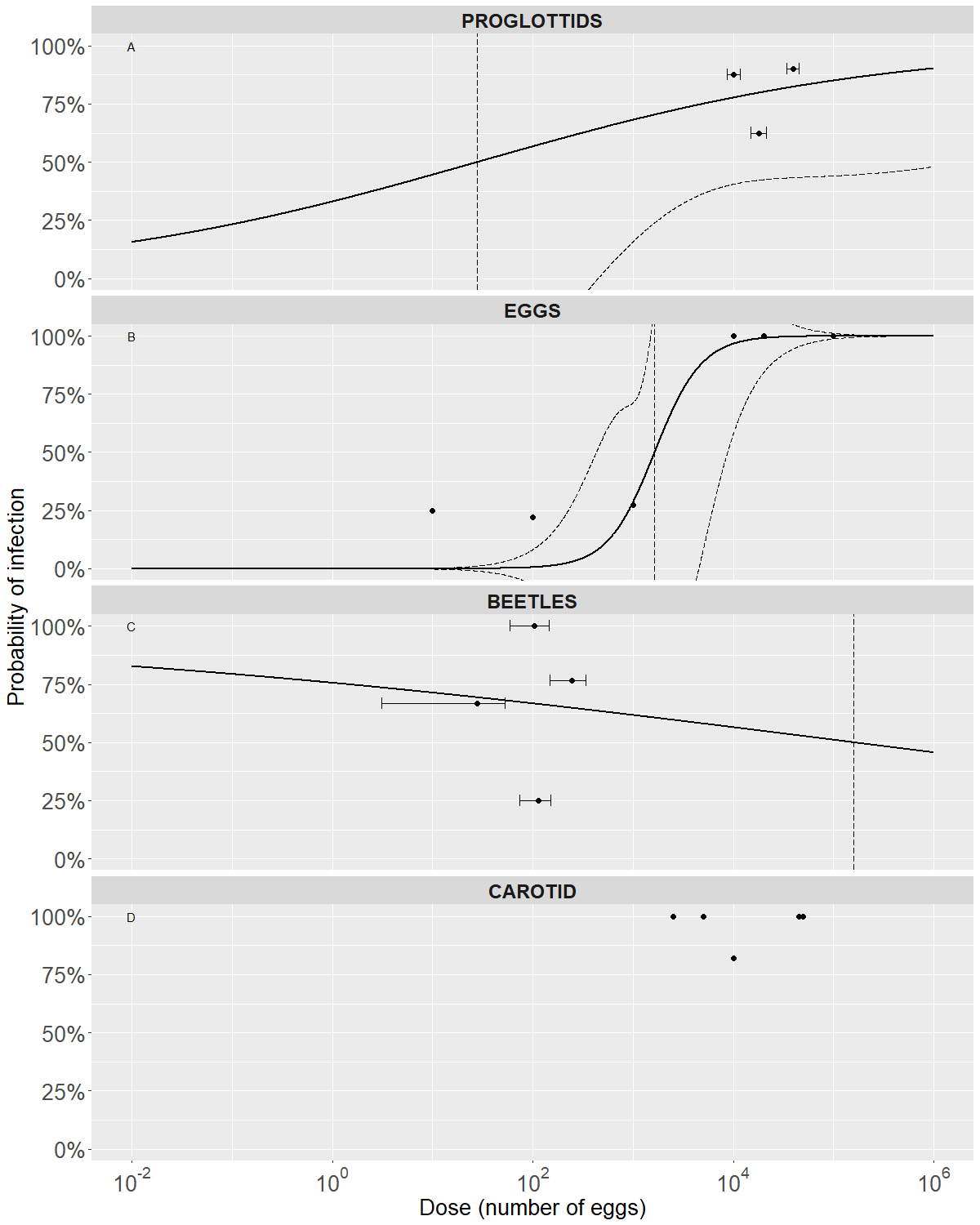


**Fig B. Two-parameter log-logistic regression dose-response relationship for the development of viable cysts by exposure pathway.** Each graph shows the median (solid black curve) and 95% range (dashed black curves) of the probability of infection as a function of dose, median ID50 infectious dose (dashed black vertical line), and the available data point with its standard deviation (“Proglottids” and “Beetles” pathways only). (A) Direct ingestion of gravid proglottids. (B) Inoculation via an endoesophageal tube of eggs placed in a gelatin capsule. (C) Direct ingestion of beetles previously fed with eggs. (D) Inoculation of activated oncospheres via catheterization of the common carotid artery.


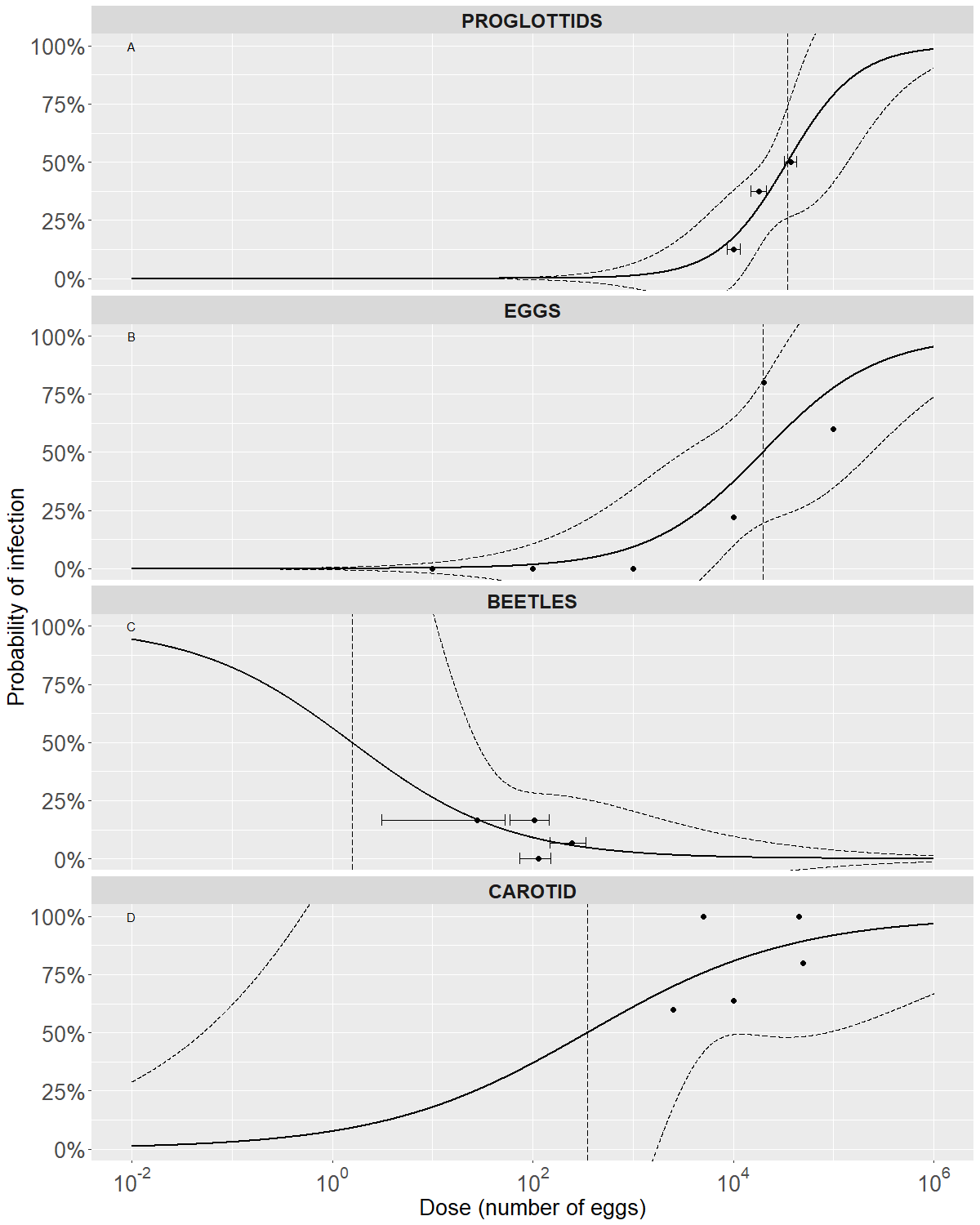


**Fig C. Two-parameter log-logistic regression dose-response relationship for the development of brain cysts by exposure pathway.** Each graph shows the median (solid black curve) and 95% range (dashed black curves) of the probability of infection as a function of dose, median ID50 infectious dose (dashed black vertical line), and the available data point with its standard deviation (“Proglottids” and “Beetles” pathways only). (A) Direct ingestion of gravid proglottids. (B) Inoculation via an endoesophageal tube of eggs placed in a gelatin capsule. (C) Direct ingestion of beetles previously fed with eggs. (D) Inoculation of activated oncospheres via catheterization of the common carotid artery.
