# Supplementary material for: Development of a dose-response model for porcine cysticercosis
Source: PLoS One. 2022 Mar 14;17(3):e0264898. doi: 10.1371/journal.pone.0264898 (PMC8920259; doi:10.1371/journal.pone.0264898)
Supplement: S6 Appendix — (DOCX) [file pone.0264898.s009.docx]

**S6 Appendix. Exact beta-Poisson model for “Proglottid”, “Beetle”, and “Carotid” pathways.**

***Supplement to* “Development of a dose-response model for porcine cysticercosis”**

$$P_{inf}\left( D \right)=1-{}_{1}{F_{1}}\left( \alpha,\alpha+\beta,-D \right)$$

**Table A. MLEs for the parameters of the exact beta-Poisson model by exposure pathway.**

| **Exposure pathway** | **α** | **β** |
| --- | --- | --- |
| **Development of any (viable or degenerated) cyst** | | |
| Proglottids | 0.066 | 0.01 |
| Beetles | 0.036 | 0.01 |
| Carotid | 0.065 | 0.01 |
| **Development of viable cysts** | | |
| Proglottids | 0.474 | 487.86 |
| Beetles | 0.180 | 0.467 |
| Carotid | 0.065 | 0.01 |
| **Development of brain cysts** | | |
| Proglottids | 0.122 | 500.00 |
| Beetles | 0.0008 | 0.01 |
| Carotid | 0.116 | 0.305 |

Legend: α, parameter alpha; β, parameter beta; Proglottids, direct ingestion of gravid proglottids; Beetles, direct ingestion of beetles previously fed with eggs; Carotid, inoculation of activated oncospheres via catheterization of the common carotid artery.

**Table B. Estimated doses necessary to cause a 1% and 50% probability (median and 95% range) for the development of any, viable and brain cysts, by exposure pathway.**

| **Exposure pathway** | **ID01** | **ID50** |
| --- | --- | --- |
| **Dose of eggs required for the development of any (viable or degenerated) cyst** | | |
| Proglottids | 0.011 (0.010 – 0.063) | 0.824 (0.693 – 23.41) |
| Beetles | 0.013 (0.011 – 0.048) | 1.08 (0.811 – 10.01) |
| Carotid | 0.012 (0.010 – 0.060) | 0.875 (0.693 – 19.09) |
| **Dose of eggs required for the development of viable cysts** | | |
| Proglottids | 9.28 (0.034 – 121.76) | 1639.47 (6.38 – 9312.35) |
| Beetles | 0.035 (0.013 – 0.617) | 8.38 (1.03 – 79.45) |
| Carotid | 0.012 (0.010 – 0.059) | 0.876 (0.694 – 19.09) |
| **Dose of eggs required for the development of brain cysts** | | |
| Proglottids | 54.94 (0.099 – 707.43) | 8.21 x 104 (1.23 x 10^4^ – 4.60 x 10^12^) |
| Beetles | 0.499 (0.074 – 8.94) | 8.04 x 10^40^ (6.42 x 10^10^ – NA^a^) |
| Carotid | 0.056 (0.012 – 1.88) | 34.77 (0.879 – 1907.20) |

Legend: ID01: estimated minimum dose to cause produce a probability of infection of 1%; ID50: estimated minimum dose to cause produce a probability of infection of 50%; Proglottids: direct ingestion of gravid proglottids; Beetles: direct ingestion of beetles previously fed with eggs; Carotid: inoculation of activated oncospheres via catheterization of the common carotid artery.

^a^ The upper limit was not computed during the simulation


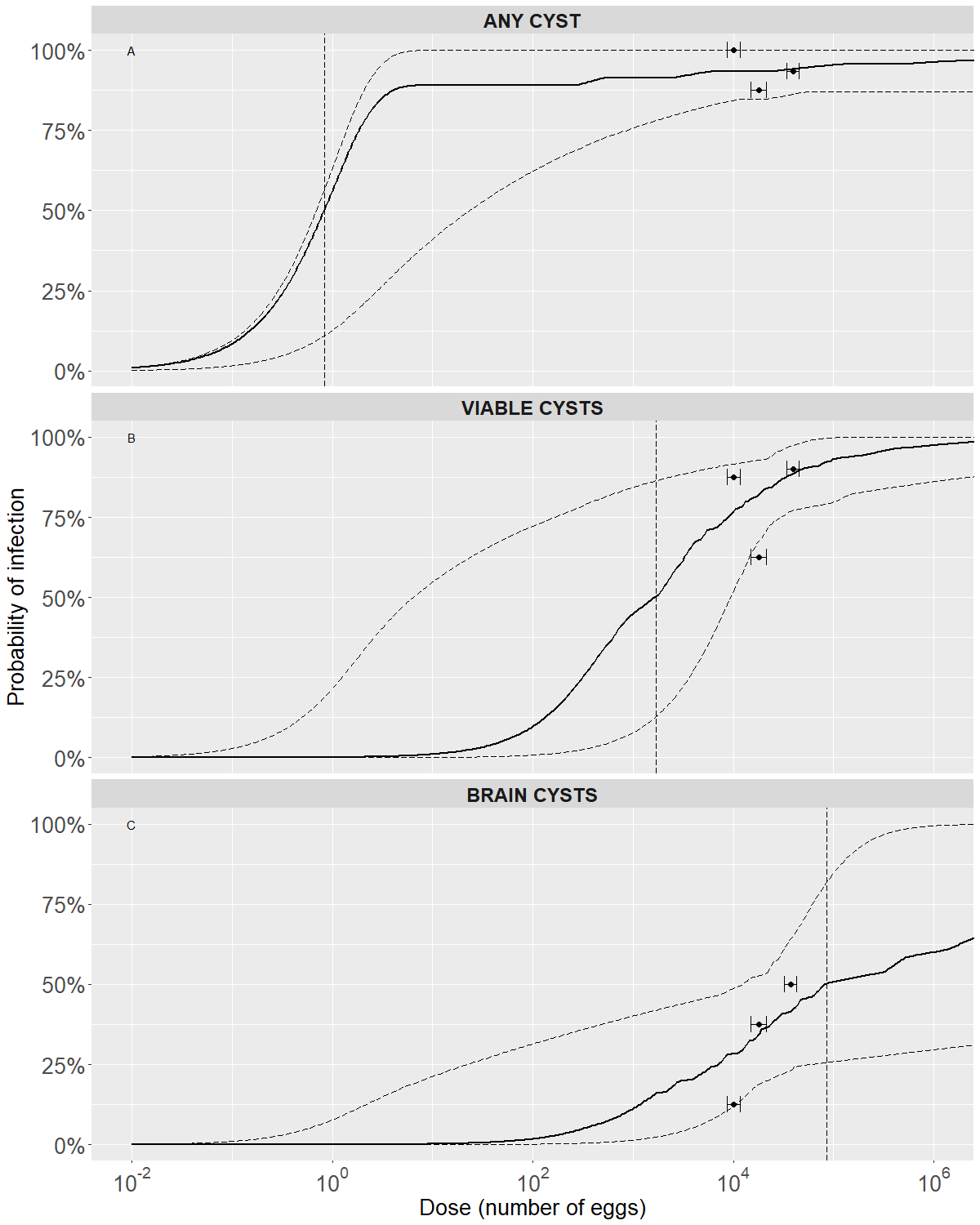


**Fig A. Exact beta-Poisson dose-response relationship for the “Proglottids” pathway by type of cyst.** Each graph shows the median (solid black curve) and 95% range (dashed black curves) of the probability of infection as a function of dose, median ID50 infectious dose (dashed black vertical line), and the available data point with its standard deviation. (A) Development of any (viable or degenerated) cyst. (B) Development of viable cysts. (C) Development of brain cysts.


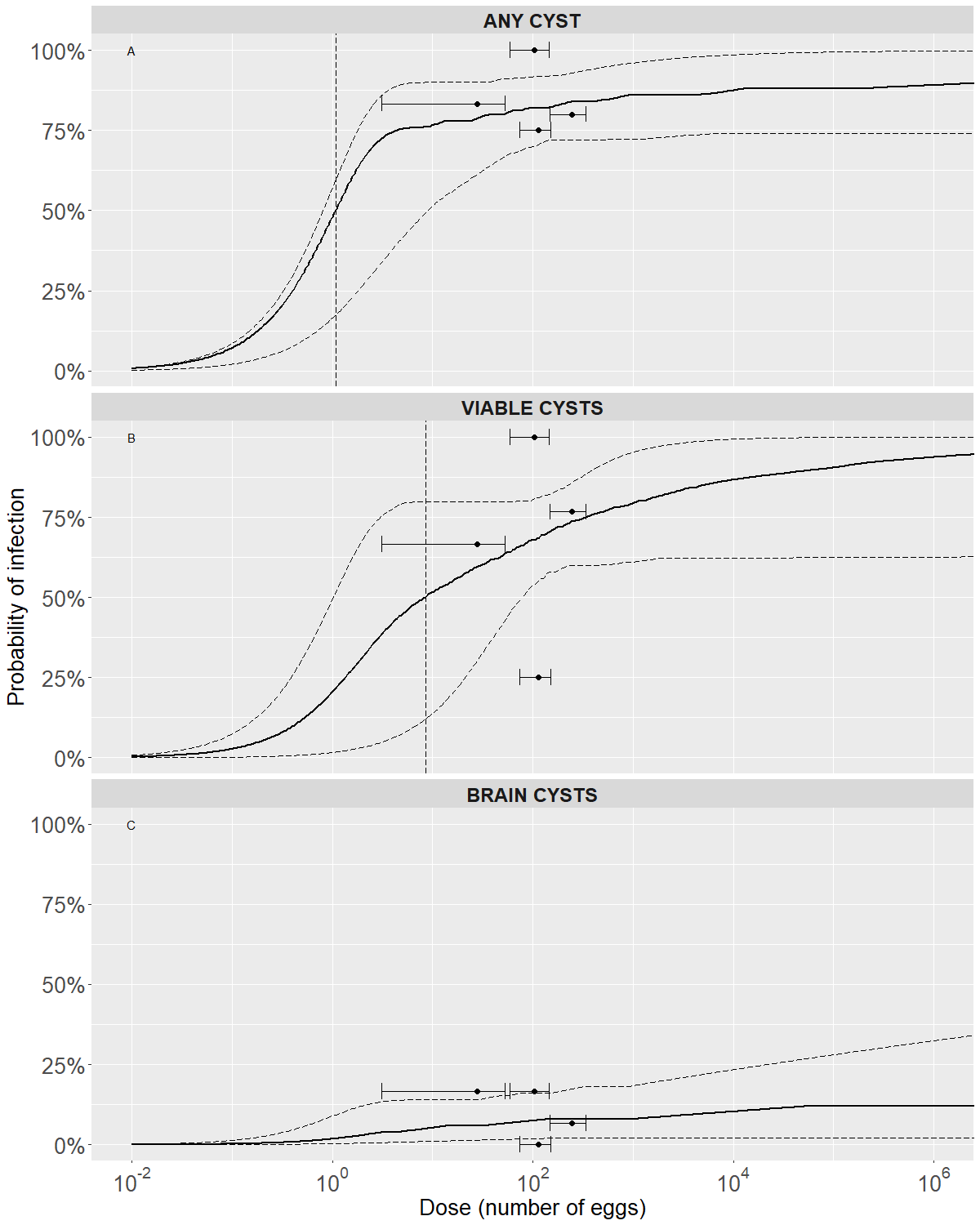


**Fig B. Exact beta-Poisson dose-response relationship for the “Beetles” pathway by type of cyst.** Each graph shows the median (solid black curve) and 95% range (dashed black curves) of the probability of infection as a function of dose, median ID50 infectious dose (dashed black vertical line), and the available data point with its standard deviation. (A) Development of any (viable or degenerated) cyst. (B) Development of viable cysts. (C) Development of brain cysts.


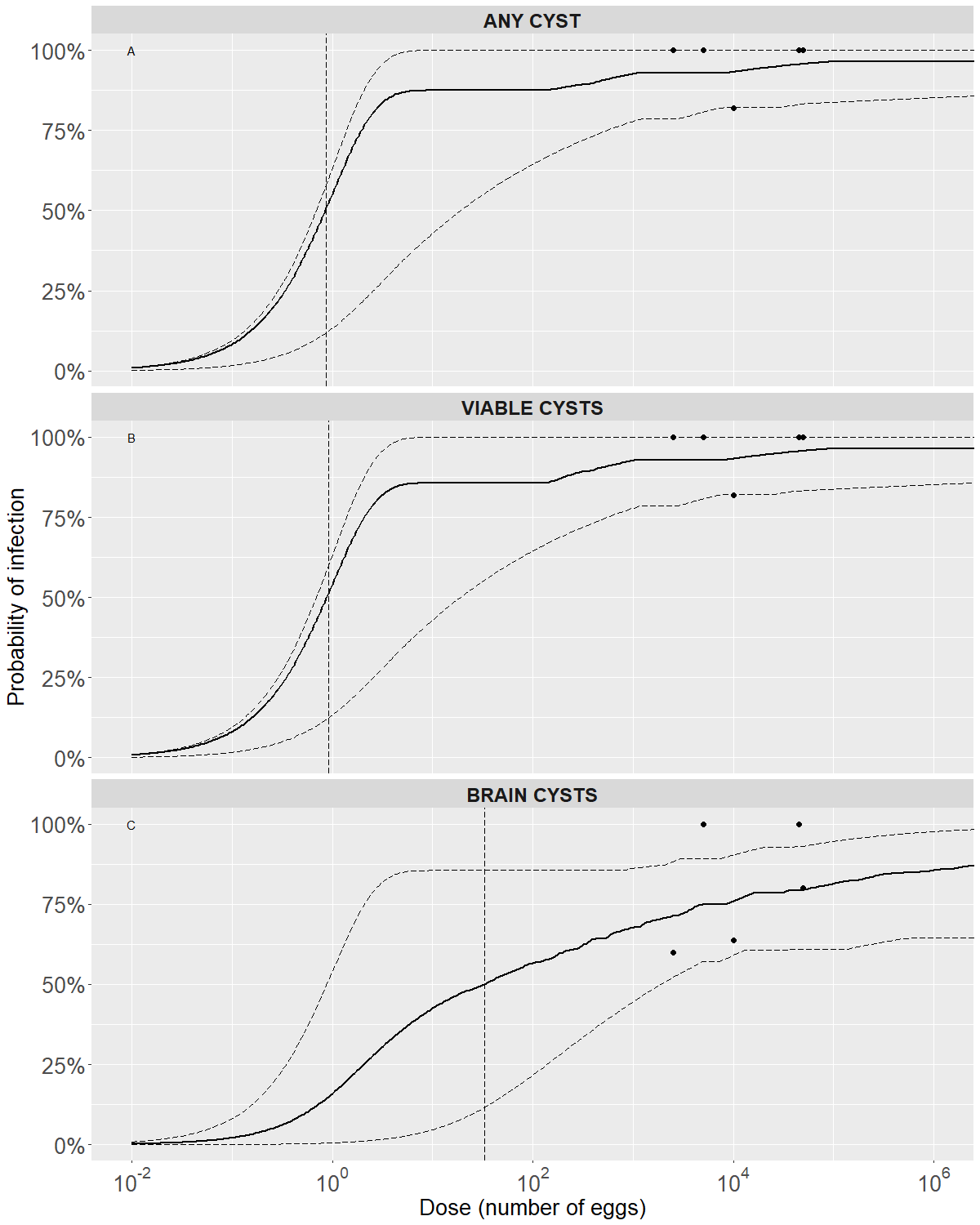


**Fig C. Exact beta-Poisson dose-response relationship for the “Carotid” pathway by type of cyst.** Each graph shows the median (solid black curve) and 95% range (dashed black curves) of the probability of infection as a function of dose, median ID50 infectious dose (dashed black vertical line), and the available data point. (A) Development of any (viable or degenerated) cyst. (B) Development of viable cysts. (C) Development of brain cysts.
