# Supplementary material for: Development of a dose-response model for porcine cysticercosis
Source: PLoS One. 2022 Mar 14;17(3):e0264898. doi: 10.1371/journal.pone.0264898 (PMC8920259; doi:10.1371/journal.pone.0264898)
Supplement: Attachment — Submitted filename: Response to Reviewers.docx [file pone.0264898.s011.docx]

Dear Editor,

We thank the reviewers and editor for their comments and suggestions that have contributed to improve the manuscript PONE-D-21-23111. We have modified the manuscript according to their suggestions in order to submit the revised version. All comments have been answered in this document and the modified sentences of the manuscript have been marked.

As requested by the Journal Requirements, the changes made to the reference list in the previous revision are specified below:

References removed:

- Massoud TF, Vinters H V., Chao KH, Vinuela F, Jahan R. Histopathologic characteristics of a chronic arteriovenous malformation in a swine model: Preliminary study. Am J Neuroradiol. 2000;21: 1268–1276.
- Negi N, Das BK. CNS: Not an immunoprivilaged site anymore but a virtual secondary lymphoid organ. Int Rev Immunol. 2018;37: 57–68. doi:10.1080/08830185.2017.1357719

References added:

- Singh BB, Khatkar MS, Gill JPS, Dhand NK. Estimation of the health and economic burden of neurocysticercosis in India. Acta Trop. 2017;165: 161–169. doi:10.1016/j.actatropica.2016.01.017
- Jansen F, Dorny P, Gabriël S, Dermauw V, Johansen MV, Trevisan C. The survival and dispersal of Taenia eggs in the environment: what are the implications for transmission? A systematic review. Parasites and Vectors. 2021;14: 1–16. doi:10.1186/s13071-021-04589-6
- De Aluja AS, Villalobos NM, Nava G, Toledo A, Martínez JJ, Plancarte A, et al. Therapeutic capacity of the synthetic peptide-based vaccine against Taenia solium cysticercosis in pigs. Vaccine. 2005;23: 4062–4069. doi:10.1016/j.vaccine.2004.11.076
- Schmidt PJ. Norovirus Dose-Response: Are Currently Available Data Informative Enough to Determine How Susceptible Humans Are to Infection from a Single Virus? Risk Anal. 2015;35: 1364–1383. doi:10.1111/risa.12323

These changes were made in accordance with the recommendations of the reviewers in the previous revision. No changes to the reference list were made in this revision.

REVIEWER #2

- The authors have addressed satisfactorily all points I raised in my previous review, with good explanations to me and by improving the manuscript accordingly. I'm pleased to say Good job :) !

Thank you for your valuable recommendations. These have improved the manuscript in a significantly way.

REVIEWER #3

MINOR COMMENTS

- It isn’t clear how relevant non-viable cysts and brain cysts are. Are pig brains ingested by humans? Are non-viable cysts of concern to the health of the human or pig in some way? It seems like more context on the transmission process would be helpful, though perhaps not strictly necessary.

Regarding the degenerated (non-viable) cysts, their relevance depends on the term of the infection in pigs. In short-term, their importance lies in the purpose of the research that requires the development of experimental infection in pigs. In contrast, the degenerated cysts mostly disappears while a reduced number calcifies in the pigs’ carcass remaining uncapable of continuing the life cycle of the parasite.

To highlight the importance of the models for the development of brain cysts, we have added the following paragraph:

Line 453: *“Despite the presence of cysts in the pigs’ brain represents a null impact on public health, the models for the development of brain cysts can also serve as reference to estimate the required doses to obtain cysts in pigs’ brain experimentally for future studies with the aim of evaluating treatment schemes for NCC using pigs as animal models. On the other hand, the model for development of brain cysts through the “Oral” pathway may be extrapolated to human NCC based on recent evidence which suggests that oncospheres are distributed to all tissues in humans, instead of being established preferentially in the brain, as was believed before [18]. However, it is necessary to demonstrate that there is no difference in the distribution of cysts between humans and pigs in order to carry out such extrapolation.”*

- Lines 53-54 – I suggest “…with ingestion of proglottids, eggs, and beetles that ingested eggs, and direct injection of activated oncospheres into the carotid artery” or similar. As written, “injected directly into the carotid artery” seems to apply to all four types of dosed material.

As suggested, we have modified the text as:

Line 53: *“…with ingestion of proglottids, eggs, and beetles that ingested eggs, and direct injection of activated oncospheres into the carotid artery.”*

- Lines 139-140 – I suggest “…a systemic infection results from a single infective dose…”

We have modified the text as suggested in the following way:

Line 139: *“…because a systemic infection results from a single infective dose…”*

- Lines 186-191 – It is not very clear how these conversions from proglottid/beetle doses to egg doses were implemented.

Thanks for letting us notice the lack of information. We have modified the idea in the following way to avoid any type of misunderstanding:

Line 186: *“In the studies that used proglottids, the infective dose for each proglottid was estimated by sampling with replacement a randomly-generated database composed of 10,000 observations following a discrete uniform distribution between 30,000-50,000 eggs for a full proglottid [13], and then linearly scaled for ½ and ¼ of a proglottid. For the “Beetles” pathway, we used the database composed by experimentally infected beetles with T. solium eggs from Gomez-Puerta et al. [37] to estimate the dose for each beetle by sampling with replacement.”*

- Line 195 – Table S2 is cross-referenced in the text before Table S1. Please check cross-referencing of tables and appendices throughout to make sure it is correct, complete, and numbered in order of appearance in the manuscript.

We have reordered the supplementary information according to the order of appearance in the manuscript.

- Line 201 – “…log-logistic regression, logistic regression, the exponential model, and approximate and exact beta-Poisson models”. “Exponential regression” is not quite right (here and throughout).

As suggested, we have replaced the term “exponential regression” by “exponential model” both in the manuscript and in S5 Appendix.

- Line 207 – Add “regression”? Although it is reparameterized in terms of ID50, it is still a generalized linear model.

We have added the term “regression” both in the manuscript and in S3 Appendix as suggested.

- Line 211 – I believe that there is a missing negative sign in front of beta_slope

As seen in “Ritz C, Baty F, Streibig JC, Gerhard D. Dose-response analysis using R. PLoS One. 2015;10: 1–13. doi:10.1371/journal.pone.0146021” (reference of the R package used to perform the dose-response analysis with this model) referenced in this section, beta_slope does not have a negative sign in the log-logistic regression function.

- Line 220 – It isn’t meaningful to describe both Pinf and r as “probability of infection”. Perhaps r is the “probability of infection from exactly one infectious agent”.

Thanks for letting us notice this point. We have modified the idea in the following way to avoid misunderstandings:

Line 222: *“This model has one parameter: the probability of at least one pathogen surviving the chain of barriers and causing an infection from any quantity of ingested pathogens (r) [42,43] (see Equation 3).”*

- Line 223 – Why not use maximum likelihood consistently for all models? Is the result the same?

Granted that the exponential model presented in this study is a non-linear model, the relationship cannot be linearized by transformation of the response variable or the explanatory variable (or both). Therefore, the parameters of this model only can be estimated by non-linear least squares rather than by maximum likelihood estimation.

- Figure 1 – The resolution of this figure is very poor

The resolution of Figure 1 has been improved as requested according to the submission guidelines for figures.

- Lines 255-259 – It isn’t clear how R^2 is being calculated for the non-regression models. I didn’t look at the R scripts closely enough to figure it out. If all models are fit by maximum likelihood, the Akaike information criterion is a simple model comparison tool for non-nested models such as these.

We have modified the following sentence in order to avoid any type of misunderstanding:

Line 259: *“For both beta-Poisson models, only the median probability of infection resulting in the posterior distribution was considered and compared to observed data to develop these calculations.”*

Regarding the use of AIC, not all the models were fitted by MLE as can be seen in the Exponential model which was fitted by non-linear least squares due to not being a linear model. Besides, the coefficient of determination (R^2^) and the Sum of Squared Errors of Prediction (SSE) are valid calculations to estimate the variation between the predicted and the observed outcomes in these models.

- Line 316 – Pooling data in this way presumes that all three scenarios share the same dose-response model. It is possible to use a likelihood ratio test to determine whether unpooled analysis (e.g., a unique set of parameter values for each scenario) provides a significantly better fit than pooled analysis (e.g., having a shared set of parameter values common to all three scenarios). Alternatively, you could compare the AIC of the pooled data model with the AIC for the three models with unpooled data.

As explained in Line 265, the purpose of pooling the data (“Proglottids”, “Eggs” and “Beetles” pathways) to build the “Oral” pathway was not necessarily the obtainment of a model with a better fit but a robust model with less uncertainty across the range of doses assessed which represents the natural infection pathway of the disease.

Due to the increasement of observations in this model, the “Oral” pathway does not have better fit scores when comparing the Sum of Squared Errors of Prediction and the Coefficient of Determination calculated with the three individual models in most of the scenarios (development of any, viable and brain cysts) (see S1 Appendix, we have added the fit scores of the exact Beta-Poisson model for the “Oral” pathway). However, this model is more robust than the other three pathways and it is evidenced in the uncertainty presented in Figure 2 across the range of observations.

For these reasons, we are not considering evaluating whether the “Oral” model provides a better fit than the individual pathways (“Proglottids”, “Eggs” and “Beetles” pathways).

- Table 3 – It is typical to report maximum likelihood estimates of parameters rather than medians.

Thanks for letting us notice this point. We have replaced the medians by the MLEs of parameters for both the exact and the approximate beta-Poisson models.

- Line 387 – Appendix S2

Thank you for letting us notice the mistake. We have corrected the in-text citation.

- There is a very small number of grammatical issues throughout:
- Line 60: Revise to “…each of the four…”
- Line 77: Revise to “…models have been developed.” (to clarify that such models were not part of this work)
- Line 107: Revise to “surrounding”
- Lines 158-162: It is problematic to have two colons in this long sentence, and use “operators”.
- Lines 281, 283: Revise to “cyst”

Thank you for letting us notice these grammatical issues. We have corrected the phrases as suggested.
